# Role of Brain Modulators in Neurodevelopment: Focus on Autism Spectrum Disorder and Associated Comorbidities

**DOI:** 10.3390/ph15050612

**Published:** 2022-05-16

**Authors:** Ali K. Saad, Amal Akour, Abdulla Mahboob, Salahdein AbuRuz, Bassem Sadek

**Affiliations:** 1 Department of Pharmacology & Therapeutics, College of Medicine and Health Sciences, United Arab Emirates University, Al Ain P.O. Box 17666, United Arab Emirates; 201370338@uaeu.ac.ae (A.K.S.); aakour@uaeu.ac.ae (A.A.); saburuz@uaeu.ac.ae (S.A.); 2 Zayed Center for Health Sciences, United Arab Emirates University, Al Ain P.O. Box 17666, United Arab Emirates; 3 Department of Biopharmaceutics and Clinical Pharmacy, School of Pharmacy, The University of Jordan, Amman P.O. Box 11942, Jordan; 4 Department of Chemistry, College of Sciences, United Arab Emirates University, Al-Ain P.O. Box 15551, United Arab Emirates; abdulla.mahboob@uaeu.ac.ae

**Keywords:** brain development, endogenous neuromodulators, neuroinflammation, pharmacological agents in-utero exposure, autism spectrum disorder, schizophrenia, attention-deficit hyperactivity disorder

## Abstract

Autism spectrum disorder (ASD) and associated neurodevelopmental disorders share similar pathogenesis and clinical features. Pathophysiological changes in these diseases are rooted in early neuronal stem cells in the uterus. Several genetic and environmental factors potentially perturb neurogenesis and synaptogenesis processes causing incomplete or altered maturation of the brain that precedes the symptomology later in life. In this review, the impact of several endogenous neuromodulators and pharmacological agents on the foetus during pregnancy, manifested on numerous aspects of neurodevelopment is discussed. Within this context, some possible insults that may alter these modulators and therefore alter their role in neurodevelopment are high-lighted. Sometimes, a particular insult could influence several neuromodulator systems as is supported by recent research in the field of ASD and associated disorders. Dopaminergic hy-pothesis prevailed on the table for discussion of the pathogenesis of schizophrenia (SCH), atten-tion-deficit hyperactivity disorder (ADHD) and ASD for a long time. However, recent cumulative evidence suggests otherwise. Indeed, the neuromodulators that are dysregulated in ASD and comorbid disorders are as diverse as the causes and symptoms of this disease. Additionally, these neuromodulators have roles in brain development, further complicating their involvement in comorbidity. This review will survey the current understanding of the neuromodulating systems to serve the pharmacological field during pregnancy and to minimize drug-related insults in pa-tients with ASD and associated comorbidity disorders, e.g., SCH or ADHD.

## 1. Introduction

In the diagnostic and statistical manual of mental disorders-5 (DSM-5) and in 2013, autism with several related neuropsychiatric abnormalities has been classified into autism spectrum disorder (ASD) [1,2]. Genetic syndromes and idiopathic autism were also grouped together based on their shared diagnostic features of either impaired sociability, communication deficits, stereotyped behaviours, or in combination [3]. Moreover, ASD falls under a larger umbrella of neurodevelopmental diseases that share some similar symptoms, and more importantly, similar pathogenesis, evident by impaired brain development. Noteworthy, some of these disorders including schizophrenia (SCH), obsessive compulsive disorder, and epilepsy co-exist with ASD [4,5].

It has been demonstrated that pathophysiologic insults in neurodevelopmental disorders start early during prenatal neuron formation and maturation [6]. However, main symptoms will mostly appear later in life during childhood or adolescence. In parallel, underlying deficits in brain maturation and wiring are present from the first neuron formed to the complete adult brain. In fact, some autistic subjects can live seemingly normal lives despite the presence of the hidden impairment [6]. Moreover, and unlike males, autistic females usually have almost normal sociability that does not mirror the imbedded autistic brain [7]. Therefore, males appear to be more affected by ASD, and the disease strikes more severely in them [8]. While the underlying neurological deficits have been extensively studied since the recognition of ASD, much further research efforts are warranted to unveil the abnormalities in this complex disorder. Accordingly, approaches developed in studying genetic syndromes provided solid ground for understanding the underlying mechanisms for ASD stemming from genetic mutations [9]. Nevertheless, genetic factors only partially contribute to the etiology of ASD. In fact, around 90% of ASD cases are classified as idiopathic [9,10]. ASD literature can be divided in two approaches: (1) the study of the autistic adult brain with the aim to developing a “correcting” remedy for patients diagnosed with ASD, and (2) tracing the pathogenesis of the disease back to the womb to identify preventative solutions. Both of these approaches can be considered by analysing the contributions of central neuromodulator systems during development, and later when the disease symptoms are manifested. In this review, several selected studies describing the negative impact of several endogenous neuromodulators and pharmacological agents on the foetus during pregnancy, manifested in numerous aspects of neurodevelopment are discussed. In addition, this review will provide insights of the current understanding of the neuromodulating systems to serve the pharmacological field during pregnancy and to minimize drug-related insults in patients with ASD and associated comorbidity disorders, e.g., SCH or ADHD. 

## 2. Neurodevelopment 

### 2.1. Neurogenesis and Synaptogenesis 

**Figure 1:**
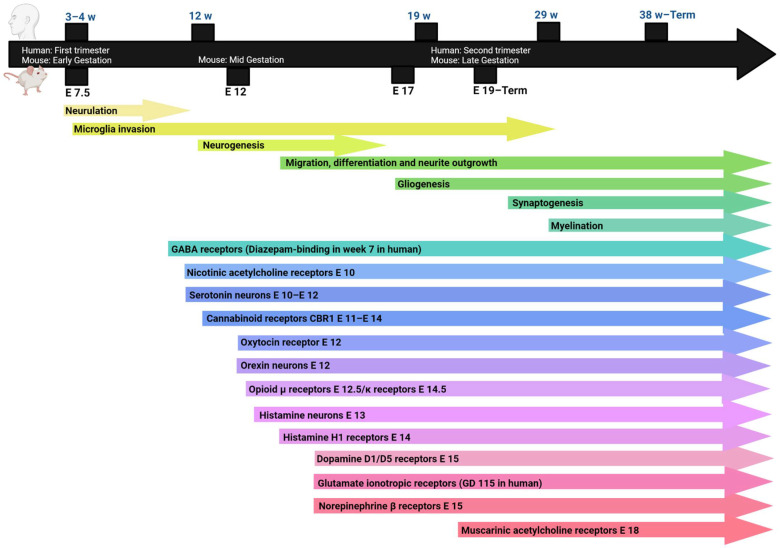
Timeline of ontogeny of neurodevelopment processes and neuromodulator systems in rodents and human in days. Top is the expected timeline in human in weeks. E = embryonic day in rodents. GD: gestation day in human. Each neuromodulator ontogeny E number is based on first expression of receptors and if possible, receptor binding ability. If receptor information is not available, neuron ontogeny is noted. Ontogeny related to detection of synthesizing enzymes, neuromodulator or mRNA levels is not considered in this graph. The use of receptor ontogeny is to predict if external pharmacological agents could have any activity. Ontogeny of the neuromodulator itself could be before or after receptor ontogeny. References for each neuromodulator are as follows: acetylcholine nicotinic [17], acetylcholine muscarinic [18], norepinephrine [19], dopamine [20], serotonin [21], glutamate ionotropic [22], GABA [23], histamine [24], oxytocin [25], opioid μ and κ receptors [26], orexin [27,28]. The figure was created in Biorender.com and was licensed for publication in this journal.

The journey of a neuronal tissue begins with the neuronal stem cells. These cells undergo proliferation and differentiation to generate neuronal and glial progenitor cells [11]. Further proliferation and differentiation of neuronal progenitor cells (NPCs) precede neuron cell formation and neurite outgrowth [11]. Some neurons are formed far from their ultimate destination, thus they need to undergo migration [12]. Neurites or projections outgrow (or sprout) to become dendrites or axons [13]. Through further maturation, axons elongate, and spines (early dendrites) protrude aided by several factors including excitatory input from neighbouring axons [14,15]. Communications, or synapses, are then formed between axons and dendrites. Chemical signalling through these synapses relies on neurotransmitters or neuromodulators stored in presynaptic compartments or vesicles until their release. Once released, they interact with postsynaptic dendritic receptors to induce excitation, inhibition, or modulation of the postsynaptic neurons [14,15]. Their receptors have different signalling pathways depending on subtypes of receptors as well as neuronal types. This process of neurogenesis and synaptogenesis starts from early gestation, with embryonic day 10 (E 10) in rodents and second trimester of pregnancy in humans (Figure 1). Further synaptogenesis and neurogenesis continue after birth and even into adulthood. For example, the hippocampus contains stem cells that upon activation can rejuvenate the neuronal formation [16].

### 2.2. Synaptic Plasticity 

It has been reported that larger excitatory axonal input during development helps in the formation of the postsynaptic dendritic spines to build mature and functional synapses [29,30]. Later, these synapses are strengthened or weakened by the same principle in a process called synaptic plasticity. Indeed, the flow of ions through excitatory postsynaptic currents (EPSC) cause a build-up of excitatory postsynaptic potential (EPSP). Shortly following high frequency stimulation, EPSP amplitude at the activated synapses increases for a longer time compared to other types of stimulation, resulting in a long-term potentiation (LTP). On the other hand, inhibitory postsynaptic currents (IPSCs) were found to induce accumulating inhibitory postsynaptic potential, which upon a low frequency stimulation is capable of yielding long-term depression (LTD). At the functional and molecular levels, both LTP and LTD represent changes in synaptic strength and participate in neurodevelopment, synaptic plasticity, learning, and memory [29,30]. Additionally, LTP and LTD can occur at different synapses in the hippocampus, cortex, amygdala, and cerebellum [29,30]. Moreover, both LTP and LTD were well studied in glutamatergic synapses, however, the same principle can occur in any neurotransmitter system including gamma aminobutyric acid (GABA) neurons [29,30].

### 2.3. Neuroinflammation 

**Figure 2:**
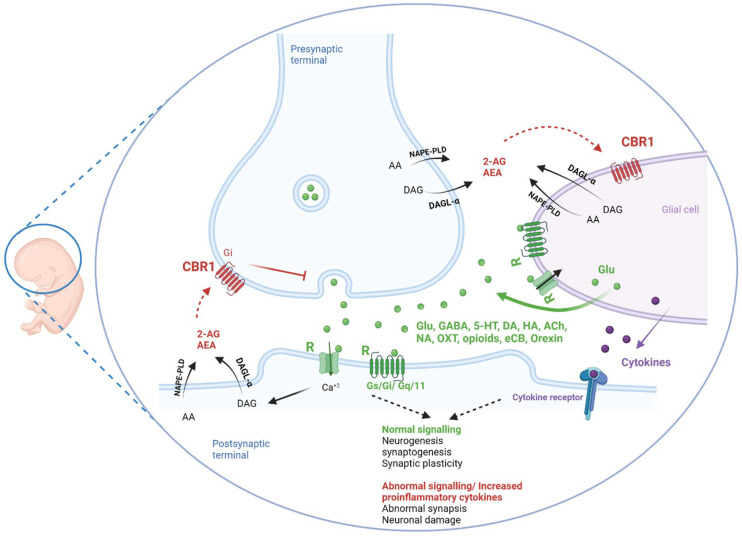
Graphic representation of synapses and microglia interaction during neurodevelopment and possible roles of neuromodulators. R—neuromodulator receptors, 2-AG—2-arachidonoylglycerol, AEA—anandamide, DAGL—diacylglycerol lipase, NAPE-PLD—N-acyl phosphatidylethanolamine phospholipase D, DAG-diaceylglycerol, AA—arachidonic acid, GLU—glutamate, DA—dopamine, NE—norepinephrine, HA—histamine, ACh—acetylcholine, 5-HT—serotonin, OXT—oxytocin. The figure was created in Biorender.com and was licensed for publication in this journal.

It has been revealed that glial progenitor cells (GPCs) are formed in parallel with precursors of neurons, namely, NPCs [31]. Upon GPC differentiation, astrocytes, that are involved in immune activation and myelin sheet formation, are developed. Immune cells migrate to the brain during early pregnancy around the timing of early neurogenesis as depicted in Figure 1 [31]. Additionally, mast cells enter the CNS during development [32]. As immune cells, mast cells and microglia have an essential role of protecting the newly developing young neurons. This is done in association with astrocytes [32,33]. Nevertheless, the involvement of astrocytes in neurodevelopment is not only limited to mere guardiancy. These resident immune cells communicate with proliferating brain cells and directly participate in different aspects of neuro- and synaptogenesis [31,34,35]. Therefore, several cytokines and mediators, which are released jointly by those immune cells, influence cell proliferation and neurogenesis. Furthermore, microglia cells were found to secrete insulin growth factor 1 (IGF-1) to aid the growth of neuronal tissues [34]. Meanwhile, and in different brain areas, microglia cells activate the apoptosis and implement phagocytic engulfment of neuronal progenitor cells to mark the end of neurogenesis during the brain maturation process. This trimming and cropping activity of microglia cells has been found to also extend to synapses. Hence, microglia cells can sense the activity of synapses and in response remove inactive synapses for the perfection of neuronal circuits in a process referred to as synaptic pruning [31]. Therefore, active synapses will then be strengthened by early LTP activity as illustrated previously under synaptic plasticity. The function of microglia cells to modulate excitatory synaptic activity during maturation to prevent excitotoxicity is confirmed [34]. Despite their essential role in proper brain maturation, microglia cells, as described by Franzen et al, are a two-edged sword [36]. Severe infection, inflammation, or other environmental or genetic disturbances can trigger abnormal activation of immune activation that starts prenatally and progresses throughout life [32,37,38]. This pathogenic neuroinflammation—opposite to the above physiological neuroinflammation—secretes abnormal levels of several proinflammatory cytokines, which at these high levels are rather destructive on the process of neurogenesis [33] (Figure 2). Overall, pathogenic neuroinflammation can be the cause, i.e., inflammatory cytokines and effect, i.e., activated to respond to insults or damage, in such scenarios [38]. ASD in fact can be manifested as an imbalance between anti- and proinflammatory cytokines, favouring pathogenic neuroinflammation has been reported by several research groups [37,39]. Furthermore, children diagnosed with ASD have been found to show increased levels of neurotensin (NT), a neuromodulator known for its effects on mast cell activation, promoting the release of numerous proinflammatory mediators and eventually triggering neurotoxicity in ASD and epilepsy [32,37]. Another related anomality was previously described by monocyte chemoattractant protein-1 (MCP-1), which functions as mast cell chemoattractant, and was found to be elevated in brains of ASD patients [32]. On the other hand, large numbers of experimental studies have revealed that these cytokines play important roles in the development of ASD [40,41]. Furthermore, peripheral upregulated inflammation (e.g. IL-6 and IL-1β) has been found to worsen autistic symptoms in mice [42]. These proinflammatory cytokines cross the blood-brain barrier and activate microglia. Dysfunctional microglia have a negative effect on synaptic pruning, influencing the signal transmission of neurons, which contributes to ASD development—a brain disorder characterized by an imbalance between inhibitory and excitatory responses [42]. In addition, peripheral blood levels of macrophage migration inhibitory factor (MIF), eotaxin-1, MCP-1, and IL-8 were also markedly increased in ASD patients compared with controls [42]. In SCH, proinflammatory cytokines were found to reduce the level of the antiapoptotic and neurotrophic growth factor (brain-derived neurotrophic factor, BDNF) gene transcription [43]. In ASD, the neuroinflammatory reaction was described as being a long-lived process that persists after birth and specifically in the cerebellum [38]. Additionally, microglia cells were found to be additionally involved in neuroinflammatory responses to early changes in gut microbiota in several neurodevelopmental disorders [44]. On the other hand, the levels of IL-38 were found to be lower in the amygdala of children with ASD and seems to inhibit microglial inflammatory mediators [41].

### 2.4. Growth Factors, Synaptic Proteins and Intracellular Calcium 

In addition to excitatory inputs, there are several proteins that were confirmed by their participation in growth of neurons and synapses, as well as in physical and morphological organization of synapses. The levels of these proteins was found to be dependent on several factors—primarily the development phase [45,46]. Examples of the proteins involved in growth of neurons and synapses include BDNF and members of the neurotrophin protein family. BDNF is produced from different cell types, including neurons, and interacts with receptors that are coupled to tyrosine kinase. The downstream signalling includes PI3K/Akt (phosphoinositide 3-kinases/Ak strain transforming) that in turn mediates cell survival and prevention of apoptosis [45]. Among other roles, BDNF was also reported to support neuron and microglia growth, neurite outgrowth, and LTP [45]. Moreover, BDNF gene expression was found to be controlled by neuron activity and consequently downstream signalling of neuromodulators [47,48]. 

Examples of the second type of structures, involved in the physical and morphological organization of synapses, are neurexin, neuroligin, and SHANK (SH3 and multiple ankyrin repeat domains proteins). They are primarily adhesion molecules and scaffolding proteins [49]. Accordingly, neuronal communication, including trans-synaptic signalling involving two families of cell-adhesion proteins, the presynaptic neurexins and the postsynaptic neuroligins, is one of the most recurrently affected pathways in neuropsychiatric disorders including ASD. Given the role of these proteins in determining synaptic function, abnormal synaptic plasticity, and failure to establish proper synaptic contacts might represent mechanisms underlying risk of ASD. 

Intracellular calcium is known to be increased by several pathways, including ion channels associated with some excitatory neurotransmitters, especially glutamate through NMDA/AMPA (N-methyl-D-aspartate / α-amino-3-hydroxy-5-methyl-4-isoxazole propionic acid) receptors. Calcium was described to exhibit many roles inside the developing neurons including regulation of gene expression, neuron stem cell differentiation, apoptosis, neuron migration, neurite growth, spine formation, receptor maturation, and microglia function [50,51]. Expectedly, impaired calcium signalling was reported to be associated with neurodevelopmental disorders including ASD [50,52]. Interestingly, the release of BDNF was found to be dependent on intracellular calcium signalling [53]. Finally, it is common knowledge that intracellular calcium, through voltage gated calcium channels, is needed for any vesicular presynaptic release of any type of neuromodulators. 

## 3. Endogenous Neuromodulators and Pharmacological Agents in Brain Development

Upon complete maturation of the brain in adulthood, neuronal systems and circuits are formed with complex synaptic interaction across brain regions. Neuromodulators at these synapses extensively participate in brain functions such as arousal, attention, cognition, emotions, and memory. Nevertheless, neuromodulators appear early in the prenatal brain before the outgrowth of any synapses [54]. Their receptors appear in the neuronal progenitor cells and many studies demonstrated their involvement in the neurodevelopmental process from neurogenesis to synaptic construction. Their receptors and functions, however, may differ between the developing and the mature brain [55]. By way of illustration, GABA_A_ receptors are excitatory in the early brain development then shift to inhibitory later [55,56]. Furthermore, glutamatergic NMDA receptors action prevails in the foetal brain. Nevertheless, glutamate effect is mediated by AMPA and kinase receptor currents later in the developed brain [57]. Figure 1 illustrates the estimated ontogeny of several neuromodulators or their signalling systems in rodent brains that coincide with the early neurogenesis. Additionally, some neuromodulators were found to serve as communication pathways between immune cells and developing neurons as depicted by Figure 2.

ASD and related neurodevelopmental disorders have both genetic and environmental components in their pathogenesis. These components will ultimately converge into deficits in one or more neuromodulator signalling systems. Table 1 summarises examples of these environmental insults sorted by the affected neuromodulators. These genetic mutations afflict genes that encode for synaptic proteins or signalling pathways. Therefore, either the synaptic structure or function was found to be disrupted. The ultimate consequence of this disruption in synapses was reported to be altered or compromised neuromodulator systems that utilize these synapses to participate in overall brain function. Some of these mutations affect proteins that are not specific to certain synapses and therefore different neuromodulator signalling pathways are concerned. For example, the SHANK3 gene is mutated in some genetic and idiopathic forms or models of ASD which display deficits in signalling pathways of numerous brain neurotransmitters including dopamine, GABA, and glutamate [58,59], as SHANK3 belongs to a family of scaffolding proteins that are connected to the postsynaptic membrane to participate in synaptic signalling [60].

### 3.1. Neurotransmitters 

#### 3.1.1. Excitatory/Inhibitory Balance (Glutamate/GABA)

Brain homeostasis and function relies mainly on excitation/inhibition balance maintained by glutamatergic pyramidal neurons and GABAergic interneurons [91,92]. Furthermore, previous reports revealed that excitation/inhibition balance is maintained through compensatory mechanisms at the level of synaptic numbers and synaptic strength [93]. For example, inhibition of GABAergic synapses by bicuculline resulted in downregulation of excitatory currents to prevent an over-excitation status of cells [93]. Excitation/inhibition balance was also found to differ with age, brain maturation, and regions [93]. For example, mature cortex has a ratio of 80 to 20% excitation/inhibition [91,93]. Additionally, GABA is the major inhibitory neurotransmitter in the adult brain, however, it has an excitatory effect in the developing brain potentiating NMDA effect [21,55,56].

Interestingly, glutamate and GABA appear early in the embryonic life, indicating their role in cell proliferation, neurogenesis, and synaptogenesis [56,91,94,95]. Loss of functional mutation in a subunit of NMDA receptor, a receptor that was identified in severe ASD, alters neuronal differentiation [96]. Consequently, exposure to drugs that affect GABA/glutamate receptors during pregnancy can therefore interfere with brain maturation before affecting any excitatory/inhibitory balance later throughout development [56,91]. Several drugs, e.g., benzodiazepines or valproate, if administered during pregnancy, were found to predispose offspring to neurodevelopmental diseases, e.g., ASD [91,97]. Furthermore, alcohol consumption by pregnant women was found to depress GABA_A_ receptor in an early stage of pregnancy, explaining the impaired growth of brains of offspring [55]. Likewise, in utero exposure to mGluR5 (metabotropic glutamate 5 receptor) or NMDA modulators (e.g., amantadine or ketamine) was reported to significantly increase the risk for ASD or other developmental disorders in neonates [98,99]. Noteworthy, the impaired excitatory/inhibitory balance was found to have its roots in every neurodevelopmental disorder including SCH, ASD, and epilepsy, to name a few [92,93]. Therefore, reduction in GABAergic neurotransmission or increment in glutamatergic circuits has been implicated in several neurodevelopmental disorders [93,100]. Nevertheless, reduction in glutamatergic function or hyperfunction of GABAergic neurotransmission can adversely affect neuron maturation and synaptogenesis. However, abnormal elevation in glutamatergic neurotransmission was found to induce neurotoxicity and consequently neurodevelopmental disorders including ASD and SCH [101,102]. On the other side, neuroinflammation could have a bidirectional role in this situation, namely, it may remove excess glutamate and prevent damage, or activate microglia cells and astrocytes that may release glutamate and proinflammatory factors with increased excitotoxicity, oxidative stress, and neuronal damage [32,101,103]. However, an appropriate amount of excitation was found to be necessary for brain maturation. Therefore, collective effects of NMDA glutamate receptors and early excitatory GABA_A_ receptors were found in previous studies to mediate normal development of the brain [21,55,56]. Furthermore, hypofunction of glutamate receptors (AMPA, NMDA, and mGluR_1-_5) was found to be associated with impaired neuronal differentiation, maturation, synaptogenesis, and either synaptic plasticity, LTP, or in combination [56,104]. Such hypofunction was also reported to constitute a predisposition to neurodevelopmental disorders such as SCH and ASD [104]. 

Moreover, synaptogenesis and neurogenesis were found to continue after birth and in the adult brain in certain areas including the hippocampus [105]. Indeed, hippocampal LTP/LTD ratio was mostly found to depend particularly on glutamate signalling through NMDA receptors, signifying the effect of hypofunction of these glutamate receptors in impairing synaptic plasticity [106,107]. Therefore, several NMDA antagonists including phencyclidines, dizocilpine and ketamine were found to induce psychosis in numerous preclinical and clinical experiments [108,109]. Likewise, GABAergic hyperfunction was described to affect synaptogenesis and plasticity [110]. Accordingly, excitotoxicity was found to be injurious in the mature brain in several neurodevelopmental disorders such as epilepsy and ASD [106]. Remarkably, lower ability of NMDA receptors to activate GABAergic inhibitory interneurons was described to be adopted by NMDA hypofunction theory of SCH [111], demonstrating that there is an overall hyperexcitation between both neurotransmission systems [108]. In further support of the latter observations, reduced GABAergic signalling was well-documented in patients diagnosed with SCH [100]. Moreover, high chloride ion concentration inside immature neurons was found to drive GABAergic effect toward excitation before the shift in ion gradient later in the developed brain [112]. Interestingly, patients diagnosed with SCH were found to exhibit an altered expression of regulatory machinery that controls chloride gradient rendering GABA less inhibitory in the adult brain [112,113]. Further contraindications arise when both hyper- and hypoexcitability coincide. In maternal valproic acid-induced ASD in rats, elevated NMDA function was shown to reside in the young brain for some time before transforming into deficit in NMDA signalling and LTP in the adult brain [106]. Additionally, valproic acid was shown to induce ASD prenatally, yet it constitutes one of the treatment modalities of ASD and co-morbidities later in life [114,115]. Taken together, a well-balanced excitation is necessary for brain maturation and its proper functions. 

#### 3.1.2. Dopamine 

The neurotransmitter dopamine and dopaminergic receptors appear early in the prenatal brain before the outgrowth of any synapses [116]. Moreover, once the dopaminergic neuronal system is formed, it undergoes changes until adulthood critical for development [116]. Interestingly, dopaminergic system maturation during adolescence coincides with onset of neuropsychiatric disease and substance abuse during this period [116]. Maturation of the excitatory/inhibitory balance in the frontal cortex was also found to be associated with dopaminergic neurotransmission, specifically dopamine D1 receptors [116]. The striatum and later the cortex are the earliest areas to receive dopaminergic innervation, and their development is significantly altered by factors influencing dopaminergic neurotransmission [116,117,118]. Dopamine signalling was reported to participate in several aspects of neurogenesis, neuron migration, and synaptogenesis [116,119], and its effect varies with receptor subtypes, brain regions, and age [116,119,120]. Furthermore, brain levels of dopamine have been found to influence neurogenesis, neuron migration, and synaptogenesis, since high or low dopaminergic signalling results in impaired plasticity of dopaminergic receptors [120]. This modulatory role of dopamine can be further highlighted by the opposite activities of neuroleptic and antipsychotic drugs in the developing versus developed brains. Consequently, exposure to different antipsychotics in the prenatal brain was reported to alter synaptogenesis while the same agents constitute the main treatment of ASD and SCH [55,119]. Overall, genetic, or environmental perturbation in the dopaminergic neurotransmission system has been associated with an array of neurodevelopmental disorders including ADHD, SCH, and ASD [120]. 

#### 3.1.3. Serotonin 

The neurotransmitter serotonin (5-HT) has an important role in neurodevelopment due to many reasons, including that (1) 5-HT is a trophic factor implicated in neural progenitor cell proliferation, synaptic plasticity, synapses, and circuit formation; (2) 5-HT was found to be supplied by the placenta in early pregnancy; and (3) that serotonergic neurons were found to be the first neuronal system to project to the forebrain in the second trimester in human pregnancy [121,122,123,124]. Furthermore, there is a period of 5-HT synthesis surge in the brain during childhood, and serotonin neuron projections and receptor expressions in the brain vary with age as a result. Denervation of serotonin neurons of an area in the mature brain was described to lead to damaged synapses and de-maturation of that area [121,122,123,124]. Noteworthy, SCH, Down’s syndrome, and ASD are neurodevelopmental diseases that were found to share the pathological basis of impaired serotoninergic neuron functions [121,122]. Interestingly, low level of lead exposure in neonates was reported to cause abnormality in the developed brain, due to damaging serotoninergic neurons [125]. Importantly, the well-known drug class of selective serotonin reuptake inhibitors (SSRIs) target serotonin transporters and thus, are capable of increasing the levels of 5-HT in the synaptic cleft [123]. Therefore, postnatal (through breastfeeding) exposure to SSRIs was found to adversely alter neurodevelopment and predispose neonates to ASD-like symptoms [123]. Bearing in mind that SSRIs are one of the most used treatment options for ASD and in similarity with valproic acid, which increases the risk of (or induce) ASD in the prenatal life but is a therapy in adulthood [126,127]. Furthermore, brain 5-HT levels were found to be disturbed during brain development in patients diagnosed with ASD compared to neurotypical individuals [123,124]. In this regard, a correlation was found between serotonin receptor subtype 7 (5-HT7R) and neurodevelopmental disorders including ASD, fragile X syndrome, and Rett syndrome. Therefore, modulators of 5-HT7R were found to improve altered behaviours in animal models and also to affect neuronal morphology via the 5-HT7Rs signalling pathway, signifying 5-HT7Rs to be a potential therapeutic target for the treatment of neurodevelopmental disorders [128]. 

#### 3.1.4. Acetylcholine

The neurotransmitter acetylcholine, through muscarinic and nicotinic receptors, was also found to have a trophic role in many cell types including neurons [129]. In addition, acetylcholine was described to regulate neuronal circuits in the developing brain [130,131]. Accordingly, nicotinic receptors were found to mediate postnatal change in GABA receptors from excitatory to inhibitory maintaining balanced excitation [131]. Hence, deficits in cholinergic neurotransmission were observed in several neurodevelopmental disorders including ASD, ADHD, Tourette disorder, and SCH [132,133,134]. Furthermore, cholinergic deficits were linked to spontaneous arousal fluctuation, an early biomarker of ASD before the actual onset of symptoms [135]. On the other hand, elevated acetylcholine levels during neurodevelopment were reported to pose the same risk as deficiency. Notably, exposure to different organophosphates, synthetic compounds which are capable of blocking the acetylcholine metabolizing cholinesterase enzyme, was directly associated with several neurodevelopmental diseases [136]. Similarly, maternal inflammation was reported to predispose the offspring to ASD through incrementing the amount of cholinergic neurons in the foetal brain [137]. 

#### 3.1.5. Norepinephrine

The locus coeruleus (LC) contains noradrenergic cell body clusters that send axonal extensions to a wide range of brain areas [19]. Pattern of LC neuron firing differs with age, as described above, partly because of postnatal impact of α_2_ receptors, which are auto-receptors. Both α- and β-adrenergic receptor subtypes were shown to be involved in growth, maturation, and formation of neurons and synapses through different signalling pathways [19]. Altered dendritic formation coupled to impairment in extracellular signal-regulated kinase (ERK) pathways, a downstream of α_2_ receptor activation, was previously observed in autistic Black and Tan Brachyury (BTBR) mice [19]. Abnormal α/β receptor signalling, polymorphism, or early exposure of their modulators, was linked to psychosis, cerebral palsy, ADHD, and ASD [19]. Postnatally, noradrenergic signalling was documented to moderate the modulation of attachment learning within the first weeks which influence behaviour later in adulthood [19]. The importance of this finding is that attachment deficit is an early sign of ASD [138]. Furthermore, disturbances in adrenergic system were found to perturb signalling of other neuronal systems such as dopamine and serotonin that are co-players in normal brain maturation [19]. Noteworthy, adrenergic neurons have a role in the modulation of microglia maturation and activity later in life, which may impact neuroinflammation and synaptic plasticity [19,139]. This is quite relevant as neuroinflammation was described to be one of the principal aetiologies in the pathogenesis of neurodevelopment disorders including ASD [140].

#### 3.1.6. Histamine

For the brain neurotransmitter histamine, two sources of histamine in the brain, namely, neurons and mast cells, have been reported [141]. The histaminergic neurons were reported to originate early at E 13 in rodents, equivalent to second trimester in humans [141]. Early contribution of histamine to neurogenesis was described to be maintained by histamine supply from other neurons (i.e., noradrenergic) and mast cells [142]. In addition to neurogenesis, histamine was found to meditate synaptogenesis, synaptic maturation, synaptic plasticity, and neural circuit formation [142]. Interestingly, a previous preclinical research showed that induced histaminergic dysfunction was postulated as a mechanistic cause in valproic acid-induced ASD in a zebrafish model [143]. Histamine interacts with four different histamine receptor subtypes, namely, H1–H4 receptors which were reported to exhibit different roles in neurodevelopment. Interestingly, H3 heteroreceptors as mentioned above were described to control the release of histamine, as well as glutamate and several other neurotransmitters [142]. Moreover, histamine was found to interact with the polyamine binding site on NMDA receptors that are implicated in synaptic plasticity [141]. By interaction with H3 receptors, histamine shows many converging roles in neurodevelopment [142]. Notably, antagonists/inverse agonists targeting H3 receptors were suggested as a possible and promising pharmacological treatment that may tackle the underlying pathogenesis of several neurodevelopmental disorders, e.g., ASD in addition to symptomatic alleviation [126,127,144,145,146,147,148]. However, the success with these agents has been limited thus far to preclinical studies, as there are isoforms of H3Rs with different distributions that contribute to the varying or rather conflicting effects of H3R antagonists/inverse agonists [126,142,149]. Interestingly, mast cells being a non-neuronal source of histamine in the brain were reported to add to the multifaceted roles of histamine in neurodevelopment. Accordingly, crosstalk between neurons and immune cells was found to be essential for neurodevelopment despite the involvement of abnormal neuroinflammation in many neurodevelopmental diseases [150]. Indeed, histamine was reported to facilitate the release of neurotrophins (e.g., glial cell line-derived neurotrophic factor, GDNF) and several other anti-inflammatory factors (e.g., interleukin-10, IL-10) from astrocytes and microglia, preventing simultaneous proinflammatory factors such as transforming growth factor beta (TGF-β) [141,142,151]. However, histamine was also reported to be capable of facilitating proinflammatory responses of immune cells in the brain through H1 and H4 receptors [151]. Noteworthy, H1–H3 receptors are expressed in neurons as well as astrocytes, microglia, and mast cells [142]. However, the H4 receptor expression profile and possible role in neurodevelopment has been limited barely to only immune cells [142,152].

### 3.2. Neuropeptides in Brain Development 

#### 3.2.1. Oxytocin

The neuropeptide oxytocin and neurodevelopment are relevant topics in obstetrics since labour is facilitated by oxytocin administration. Additionally, concentration of oxytocin in the maternal blood was found to be increased naturally during the perinatal time for the same purpose. In this regard, four plausible questions arise: (1) can oxytocin be transferred from mother to foetal blood and more importantly to foetal brain? (2) is there any oxytocin receptor expression in the foetal brain? (3) is there any oxytocin production inside the immature brain? and finally, (4) how does oxytocin affect neurodevelopment? The following paragraphs will be devoted to answer these questions. 

Oxytocin can be supplied to the foetus via the placenta to its blood [153]. Oxytocin’s entry into the brain exploits the state of immaturity of the blood brain barrier (BBB) in this period, as it was observed that immature BBB is permeable to oxytocin [153]. The significance of the latter finding is that it offers an explanation for the clinical observation of increased neurodevelopmental disorders in children delivered through oxytocin-facilitated labour [153]. Monks et al. reviewed several studies that concluded such observations. Therefore, natural oxytocin is needed in this period for proper brain development as well. In a previous preclinical study, null mutant male mice were found to differ from another null mutant mice, and this difference was dependent on the presence of oxytocin in their mothers. Null mutant mice delivered by null mutant dams were described to be more aggressive compared to their conspecifics delivered by heterozygous dams. Additionally, their behaviours were not affected if they were cross-fostered by wild-type dams [154]. Therefore, direct maternal transplacental transfer of oxytocin is important for adult behaviours. Moreover, perinatal oxytocin impact on neurodevelopment can be attributed to different factors. First, perinatal oxytocin contributes to the shift of GABAergic signalling to inhibitory before birth in rodents and thereby it modulates excitatory/inhibitory balance [155,156]. Second, oxytocin confers beneficial activities in maturation, neurogenesis, and synaptogenesis of hippocampus neurons in the newborn [156,157]. Finally, oxytocin prevents neuroinflammation during the perinatal period [158]. Taken together, perinatal oxytocin was found to be essential for shaping adult behaviours, but moderate levels are required. Noteworthy, oxytocin receptors can couple to Gi or Gq/11 and generate a plethora of downstream activities depending on the oxytocin levels that are present [159]. 

Oxytocin receptors were detected as early as E-12.5 in the mouse brain; however, oxytocin itself was not expressed until postnatal day 2 [25,154]. Excitingly, females displayed oxytocin mRNA at E 16.5 and as compared to males, where the mRNA appears much later [154]. Although it is not known when oxytocin will be expressed, it can be assumed that at least there is a gender specific development of oxytocinergic neurons in a reductionist’s viewpoint [154]. More broadly, it is also reasonable to assume a role of oxytocin in gender differences in neurodevelopment and consequently adult behaviour [154,160]. Additionally, the density of oxytocin receptors was found to be significantly higher in the female foetal brain [154]. Notably, oxytocin was found to regulate social behaviours and social recognition memory besides other roles [154,156,161,162,163]. Therefore, abnormalities in oxytocin levels would produce more sociability-impairing consequences in males than females. Interestingly, recent studies found a strong correlation between higher oxytocin receptor gene methylation (i.e., silencing) and either reduced IQ, social interaction, or in combination, scores in patients with neurodevelopment disorders [164,165]. 

Despite the fast move to clinical trials with oxytocin, little attention has been paid to the possibility that the oxytocin system in the brain is perturbed in these disorders and to what extent such perturbations may contribute to social behaviour deficits. However, large-scale whole-exome sequencing studies in subjects with ASD, along with biochemical and electrophysiological studies in animal models of the disorder, indicated several risk genes that have an essential role in brain synapses, suggesting that deficits in synaptic activity and plasticity underlie the pathophysiology in a considerable portion of these cases. Moreover, oxytocin has been repeatedly shown, both in vitro and in vivo, to alter synaptic properties and plasticity and to modulate neural activity in circuits that regulate social behaviours. Together, these observations concluded that failure of the oxytocin system during early development, as a direct or indirect consequence of genetic mutations, may influence social behaviours by altering synaptic activity and plasticity [111].

#### 3.2.2. Opioids 

Endogenous opioids consist of endorphins, enkephalins, and dynorphins [166]. To varying degrees, they all interact with three types of opioid receptors, namely, mu, delta, and kappa. All these receptors are associated with Gi proteins. Furthermore, opioid receptors were found to participate in pain perception between the brain and spinal cord [167]. Additionally, they were reported to mediate emotions and behaviours such as distress (emotional pain), anger, anxiety, sadness, motivation, and social affiliative actions [168]. Moreover and in rodents, endogenous opioids and their receptors were reported to be expressed at different time points during embryonic days, namely, between embryonic days 13 and 21, with delta receptors being the late receptor subtype to appear [169]. There is an interesting hypothesis reported early in the literature that assumed opioid excess in the brain is a cause or a factor of autistic features. A recent case report hypothesized a “high opioid tone” group of autistic patients [170,171,172]. However, the use of opioid receptor antagonist naltrexone to overcome such excess resulted in a significant reduction in hyperactivity without any improvement in the core symptoms of ASD [173]. Furthermore, details about the exact roles of endogenous opioids during development are scarce and mostly come from early literature. Yet, there are several reasons that support their involvement in neurodevelopment. First, preclinical and very recent clinical studies indicated that in utero exposure to exogenous opioids, such as morphine, significantly alter neurodevelopment of the neonates [174,175]. Mechanistically, prenatal exposure to an opioid agonist may decrease myelination and augment apoptosis of neurons and microglia [176,177]. In addition, opiates were found to decrease proliferation of cerebellar granular neurons in vitro and as expected could decrease cerebellar growth [178]. Secondly, a previous in vitro study in murine neuronal stem cells suggested that δ receptors have an important contribution to neurogenesis and neuroprotection [179]. Endogenous opioids were described as being negative growth factors during brain maturation, and opioid receptor antagonist naltrexone administration during pregnancy was found to increase proliferation of brain cells and overall brain weight [180]. Moreover, the abuse of some addictive agents or drugs (ethanol, and cocaine) that have neurodevelopmental consequences was clearly associated with elevated opioid signalling in different areas of the developing brain [79,167,181]. Therefore, neurodevelopmental changes produced by the addictive drugs during pregnancy can partially be explained by an opioid receptor upregulation mechanistically influencing neuron maturation[182]. Additional important consideration is that endogenous opioids were found to induce inhibitory sequela on maturation of prenatal neurons [180,182]. In harmony with the above-mentioned preclinical and clinical observations and following prenatal addictive drug exposure, dopaminergic neuronal function was found to be modified due to heightened opioid signalling at these neurons during development [79,181]. 

#### 3.2.3. Cannabinoids 

Two main endogenous or natural cannabinoids (eCB) were identified, namely, 2-arachidonoyl glycerol (2-AG) and anandamide (AEA) [183]. Both cannabinoids were reported not to be secreted in the conventional vascular pathway such as other classical neuromodulators [183]. They are released from postsynaptic neurons—and sometimes microglia—to control presynaptic activity and release of other neuromodulators such as glutamate and GABA among others as depicted in Figure 1 [183]. Consequently, 2-AG and AEA will then bind either cannabinoid receptors 1 (CBDRs1) or CBDRs2, which are coupled to Gi proteins. It was previously reported that neurons mostly express CBDRs1, and CBDRs2 are predominately found in immune cells including microglia and mast cells [183,184]. Importantly, in utero exposure to exogenous cannabinoids was found to produce deficits in maturation of the foetal brain which are strongly associated with SCH [185,186]. Do these deficits indicate a role of eCB signalling in neurodevelopment since exogenous cannabinoids are able to bind to some other “off-target” receptors? In humans, it has been reported that CBDRs1 are expressed as early as 14 weeks of gestation (equivalent to embryonic day 12 in rodents) and gradually grow in density to adulthood [187]. Similarly, mRNA of these receptors was detected around embryonic days 11–14 in rats. Many previous studies that utilized genetic or selective pharmacological manipulations of eCBD receptors concluded the involvement of this neuronal system in about each aspect of brain development. Hereafter, Alexandre et al. nicely reviewed roles of CBDRs1 and CBDRs2 in regulating proliferation of neuronal progenitor cells, neurogenesis, migration of neurons, and synaptogenesis [188]. In ASD children, significantly low levels of expression of N-acyl phosphatidylethanolamine-specific phospholipase D (NAPE-PLD), one of the synthesizing enzymes of AEA, were found compared to normal individuals [189]. Foetal exposure to cannabinoids impacts other neuronal systems. Therefore, cannabinoids were found to alter the expression of tyrosine hydroxylase and proenkephalin which are essential in the formation of dopamine and enkephalin, respectively [190]. Furthermore, CBDRs were found to be functional at an early stage of development during the time of ontogeny of other neuronal systems [191]. Additionally, CBDRs were found to be expressed in microglia and immune cells, especially CBDRs2, and several previous studies indicated the anti-inflammatory and neuroprotection roles of cannabinoids [184,189,192,193]. In agreement with other neuromodulator systems mentioned above, exogenous cannabinoid can be used to treat some neuropsychiatric disorders despite their damaging effects during prenatal stages. These agents could improve either behavioural anomalies, neuroinflammations, or both, in several animal models of ASD [194]. In clinical trials, amelioration of core symptoms of ASD by medicinal cannabinoids was found to be less pronounced than their seizure-inhibiting activity, since seizure is one significant co-morbidity in ASD [195]. Their activity on seizure control was found to stem from their modulatory roles on the release of GABA and glutamate and subsequent control of excitatory/inhibitory balance. Interestingly, cannabidiol has recently been approved by the Food and Drug Administration (FDA) and the European Medicine Agency (EMA) for the treatment of two ASD-associated epilepsy syndromes, namely, Lennox–Gastaut and Dravet syndromes [189]. 

#### 3.2.4. Orexin 

The two subtypes of orexin peptide, namely, orexin A and B, were found to have a neuronal system in the brain originating from lateral hypothalamus and spreading to most of the CNS [196]. Orexin peptides were detected at E 18 much later than their neurons that are expanding during E 12 [27,197]. Orexin’s late appearance and slow maturation during postnatal weeks was described to coincide with their main role in the sleep/wake cycle and feeding behaviour [27,197,198,199]. Moreover, orexins were reported to exhibit neurotrophic roles and to improve LTP in the developed adult hippocampus [196]. Furthermore, orexins were found to provide neuroprotective effects after hypoxic or ischemic attacks [200]. Furthermore, and through their receptors, orexins were shown to induce pro-survival changes in cultured cortical neurons and mediated mainly through orexin receptor 2 (OXR2). These cultured neurons are harvested from Wister rat embryonic brains on E 17 [201]. Thus, it is plausible to anticipate orexins’ involvement in the maturation of the embryonic brain. However, there is limited evidence about their participation in brain development. Hereafter, orexin A was found to be lower in serum levels in untreated ADHD children [202]. In patients with Prader–Willi neurodevelopmental syndrome, low level of orexin was also reported in their cerebrospinal fluid (CSF). Accordingly, the latter findings may suggest the involvement of orexins in the symptomatology of these diseases; however, their role in the pathogenesis cannot be clearly inferred [203]. Prenatal exposure to some agents capable of altering the orexin system, e.g., nicotine or ethanol, was shown to induce neurodevelopmental changes in adulthood. Consequently, nicotine intake during pregnancy was found to increase orexin neurons and their projections to reward-mediating dopaminergic cells at the ventral tegmental area (VTA) [77]. Furthermore, in utero exposure to ethanol was confirmed in its function to perturb the development of orexin neurons [89,90]. Therefore, the orexin system can significantly contribute to the behavioural changes in the offspring due to maternal nicotine or ethanol intake. In addition, orexin was found to noticeably modulate neurodevelopment through enhancing the release of GABA and glutamate in the prenatal brain of offspring of pre-treated rats [204].

## 4. Conclusions

Neurodevelopmental disorders including SCH, ADHD, and ASD have been found to incorporate impairments in several neuromodulator systems. Theses impairments interfere with proper maturation and vital brain functions, as well as postnatal development. The cause of such impairments can be attributed to either genetic mutations, environmental insults including pharmacological agents clinically used during pregnancy, or both. However, some of these pharmacological agents can be used later in life to correct the ailment in neuromodulator systems. Overall, understanding of these neuromodulator systems from prenatal to geriatric stages is essential in searching for the possible causes and potential pharmacological interventions for the management of ASD and associated comorbidity disorders, e.g., SCH or ADHD.

## Figures and Tables

**Table 1:** The neuromodulatory effects of prenatal exposure to pharmacological agents and addictive substances in animal models.

Drug	Affected System	Preclinical Outcomes	References
Valproate	GABA Glutamate Serotonin Oxytocin	Auditory dysfunction relevant to ASD Autism-like behaviour Autism-like behaviour Autism-like behaviour Autism-like behaviour	[61] [62,63] [64,65] [66]
Diazepam	GABA	Anxiety Sex-specific changes in social interaction	[67]
Ketamine	GABA	Model of treatment-resistant schizophrenia	[68]
Selective serotonin reuptake inhibitors (SSRIs)	Serotonin Dopamine Noradrenaline Oxytocin	Neurodevelopmental changes, warning against intake during pregnancy elevated anxiety + low sociability	[69] [70]
Antipsychotics	Dopamine	Reduced spatial learning and reduced postnatal activity of dopaminergic neurons	[71,72]
Sevoflurane	Glutamate/GABA balance	Impaired learning Depression-like behaviour	[73]
Glucocorticoids	Dopamine	Altered sociability Anhedonia Depression-like behaviour	[74]
Nicotine	Dopamine Orexin	Disturbed dopaminergic activity ADHD-like behaviour Increased drug abuse	[75,76] [77]
Amphetamine	Dopamine	Altered activity of dopaminergic neurons	[72]
Cocaine	Dopamine Opioids	Disrupted development and activity of dopaminergic neurons Disrupted opioid expression in dopaminergic neurons	[72,78] [79,80]
Ethanol	Dopamine, Serotonin Acetylcholine Oxytocin Opioids Orexin	ADHD-like behaviour Depressive behaviour Cognitive deficit Impaired sociability Impairment in working memory and reversal learning Impaired attentional set shifting Deficit in social interaction and depression-like behaviour Increased likability of ethanol taste Ethanol abuse	[81,82,83] [84,85] [86,87] [88] [89,90]

## Data Availability

No new data were created or analyzed in this study. Data sharing is not applicable to this article.

## Abbreviations

2-AG	2-Arachidonoylglycerol
5-HT	Serotonin
ADHD	Attention deficit hyperactivity disorder
AEA	Anandamide
Akt	Ak strain transforming also known as protein kinase B
AMPA	α-Amino-3-hydroxy-5-methyl-4-isoxazole propionic acid
ASD BDNF	Autism spectrum disorder Brain-derived neurotrophic factor
BTBR	Black and Tan Brachyury
CBR	Cannabinoid receptor
CSF	Cerebrospinal fluid
DSM DAG	Diagnostic and statistical manual of mental disorders Diaceylglycerol
eCB	Endogenous cannabinoids
E or ED	Embryonic day
EMA	European Medicine Agency
EPSP	Excitatory postsynaptic potential
ERK	Extracellular signal-regulated kinase
FDA	Food and Drug Administration in USA
GABA	Gamma aminobutyric acid
GDNF	Glial cell line-derived neurotrophic factor
Gi	Inhibitory G-protein
Glu	Glutamate
GPC	Glial progenitor cell
Gs IGF-1	Stimulatory G protein Insulin growth factor 1
IL-6 IL-8 IL-β IL-10 IPSCs LC	Interleukin-6 Interleukin-8 Interleukin-beta Interleukin-10 Inhibitory postsynaptic currents Locus coeruleus
LTD	Long-term depression
LTP	Long-term potentiation
mGluR MCP-1	Metabotropic glutamate receptor Monocyte chemoattractant protein 1,
MIF	Macrophage migration inhibitory factor
NAPE-PLD	*N*-acyl phosphatidylethanolamine-specific phospholipase D
NMDA	*N*-methyl-D-aspartate
NPC NT OXR2	Neuronal progenitor cells Neurotensin Orexin receptor 2
PI3Ks	Phosphoinositide 3-kinases
SCH	Schizophrenia
SHANK	SH3 and multiple ankyrin repeat domains protein
SSRIs	Selective serotonin reuptake inhibitors
TGF-β	Transforming growth factor beta
VTA	Ventral tegmental area
